# Editorial: New insights into innate immune cell-based immunotherapies in cancer

**DOI:** 10.3389/fimmu.2024.1401665

**Published:** 2024-04-09

**Authors:** Anne Caignard, Mary Poupot-Marsan, Virginie Lafont, Daniela Wesch, Chiara Porta

**Affiliations:** ^1^ Institut National de la Santé et de la Recherche Médicale (INSERM) U1160, Institut de Recherche Saint-Louis Hôpital Saint Louis, Paris, France; ^2^ Centre de Recherche en Cancérologie de Toulouse (CRCT), UMR1037 Institut National de la Santé et de la Recherche Médicale (Inserm)-Univ. Toulouse III Paul Sabatier-ERL5294 Centre National de la Recherche Scientifique (CNRS), 2 avenue Hubert Curien Oncopole de Toulouse, CS53717, Toulouse, France; ^3^ Institut de Recherche en Cancérologie de Montpellier (IRCM), Institut National de la Santé et de la Recherche Médicale (INSERM) U1194, Univ Montpellier, Institut du Cancer de Montpellier (ICM), Montpellier, France; ^4^ Institute of Immunology, University Medical Center Schleswig-Holstein, Christian-Albrechts University, Kiel, Germany; ^5^ Department of Pharmaceutical Sciences, University of Eastern Piedmont, Novara, Italy; ^6^ Center for Translational Research on Autoimmune & Allergic Diseases (CAAD), University of Eastern Piedmont, Novara, Italy

**Keywords:** macrophages & neutrophils, NK cells, γδ T cells, CAR-NK, bispecific antibodies, patient avatars, organoids, MDSC

The limited number of patients achieving an effective and durable response following T cell-centric immunotherapies indicates the urgent medical need for complementary approaches. Recent insights in innate immune cell-based cancer immunotherapies are discussed in this Research Topic, encompassing several original research manuscripts unveiling antibodies, genetic engineering and other promising strategies to enhance the anti-tumor activity of macrophages, NK and γδ T cells. Additionally, reviews within this Research Topic offer a comprehensive overview of the state-of-the-art approaches for targeting specific innate cell subtypes in cancer therapy. They also discuss emerging druggable pathways poised to advance toward clinical application in the foreseeable future, and highlight current challenges, including patient avatars as an essential tool to guide clinical decision making.

As key orchestrators of a tumor-promoting microenvironment, tumor-associated macrophages (TAM) have long been recognized as promising therapeutic targets for the development of new anti-cancer therapies. In this context, the preclinical studies of Lavvy et al. indicate the activation of ChemR23 through an agonist monoclonal Antibody (mAb) as a novel potential strategy to reprogram macrophages toward a less inflammatory and immunosuppressive phenotype and to dampen triple negative breast cancer progression. Although rare and sparse compared to macrophages, cytotoxic innate lymphoid cells infiltrate solid cancers and contribute to the elimination of tumor cells. Interestingly, the studies of Campos-Mora et al. highlight a specific CD45RARO^+^CD107a^+^ NK cell subset that, after having acquired antigens from breast cancer cells through trogocytosis, returned in the blood circulation. The identification of these circulating activated NK cell subsets carrying solid tumor markers provides the rationale to argue for its use in complementing established therapies or as a theragnostic approach for solid cancer patients. For the development of effective NK-based immunotherapies, several strategies have been investigated to enhance the cytotoxic activities and prolong the half-life of NK cells. For example, Carreira-Santos et al. established a protocol to activate NK cells isolated from the blood of healthy donors with a cytokine cocktail consisting of IL-12, IL-15 and IL-18. As a result, cytokine-induced memory-like (CIML) NK cells showed increased cytotoxicity *in vitro* and a longer lifespan, valuable features for adoptive cell transfer immunotherapies. The identification of effective combination strategies to enhance anti-tumor immunity is crucial to improve the efficacy of immunotherapy. In this regard, Lutz et al. demonstrate that novel bispecific antibodies targeting the activating receptor NKG2D and the malignant B cell antigen CD20 can potentiate both antibody-dependent cell-mediated cytotoxicity (ADCC) of anti-CD38 or anti-CD19 mAbs and the effectiveness of approved bispecific mAbs directed against CD3 and CD19, suggesting a synergistic effect between NK and T cells. Another promising combination immunotherapy consists of a novel high affinity human antibody specific for the activating receptor OX-40 (BT6026) with the antibody blocking the inhibitory checkpoint PD-1. Specifically, Liang et al. show that BT6026 had an enhanced ADCC effect and a significant anti-tumor activity in an OX40-humanized mouse model of colon cancer. Moreover, when combined with an anti-PD-1 antibody, BT6026 resulted in a synergistic effect on tumor inhibition. The good safety profile observed in non-human primates further warrants additional studies of the long-term safety and efficacy of BAT6026 in clinical trials, to assess its potential as a cancer immunotherapy.

The participation of cytotoxic innate immune cells (NK, γδ T cells) in novel adoptive cell therapies using selected innate cell subtypes or chimeric antigen receptor (CAR) engineered innate cell subsets are increasing. In this perspective, Imeri et al. provides preclinical evidence of efficacy and specificity of an Interleukin-2-Receptor a subunit (IL2RA/CD25) CAR-NK-based therapy for chronic myeloid leukemia (CML), thereby indicating a new potential therapeutic option for CML patients that are in blast phase and resistant to targeted therapies. Lizana-Vasquez et al. review the studies based on CIML NK cells for cell therapy in autologous settings, while Rimailho et al. and Wang et al. discuss γδ T cells in cancer immunotherapies. Specifically, Rimailho et al. focus on the current understanding of γδ T cell biology in the context of B-cell malignancies. They describe the diversity of γδ T cells in both tissues and blood, highlighting their potential functional plasticity and anti-tumor properties as exploitable features for immunotherapeutic approaches. The review also provides a comprehensive description of the strategies to harness γδ T cells, such as activation and tumor-targeting, expansion protocols and gene modification. Additionally, it summarizes ongoing clinical trials in B cell malignancies. Along the same line, Wang et al. offer a clinically focused review that points to key gaps in knowledge and proposes strategies to harness the unique properties of γδ T cells for cellular immunotherapy, drawing insights from previous clinical trials. Moreover, the review gives an update on ongoing trials involving γδ T cells for both hematological and solid cancers and discusses strategies that have been tested or can be explored to improve anti-tumor activity and durability of γδ T cells.

The advent of immunotherapy has fostered the development of tumor models, such as organoids, organotypic tissue slice culture, organ-on-a-chip and patient-derived xenografts, in order to get insights into the cross-talk between cancer and immune cells and to test immunotherapeutic approaches. The advantages and disadvantages of each model system are discussed by Kayser et al., who highlight the need to define the rationale and requirements for their use and the importance to advance further in the development of patient avatars as a complementary tool for testing and predicting immunotherapeutic strategies for personalized tumor therapies. In addition, two original research manuscripts of this Research Topic report recent insights into the generation and utilization of these novel models to study γδ T cells in the context of cervical and ovarian cancer. In particular, Dong et al. have established patient-derived healthy and transformed cervical organoids expressing HPV16 oncogenes E6 and E7. Using bulk-RNA sequencing, they revealed differences in DNA damage and cell cycle checkpoint pathways and identified crucial molecules for γδ T cell activation. Schadeck et al. investigate the immunosuppressive effect of galectin-3 on different γδ T cell subsets using co-culture systems consisting of ovarian cancer and Vδ1 or Vδ2 T cells. Given that galectin-3 inhibits proliferation of Vδ2 T cells only, the results of this study suggest that an activation of Vδ1 T-cell proliferation, as part of a T-cell-based immunotherapy, can be advantageous due to their resistance to the immunosuppressive properties of galectin-3.

Besides boosting cytotoxic effectors, targeting myeloid cells in order to reprogram the immunosuppressive tumor microenvironment (TME) into an immunostimulatory one has emerged as a promising strategy to enhance the efficacy of cancer immunotherapy. Therefore, an in-depth understanding of the mechanisms driving myeloid-derived suppressor cell (MDSCs) generation, suppressive activities and recruitment in the TME are essential to identify effective combinatorial strategies for tumor immunotherapy. In this context, Zhao et al. summarized current understanding of functional and regulatory mechanisms of MDSCs within the TME, along with recent insights into therapeutic strategies targeting MDSCs in combination treatments for cancer patients. Additionally, Zhu et al. report on the role of neutrophils and neutrophil extracellular traps (NETs) in the initiation and progression of hepatocellular carcinoma. They also review studies conducted on NETs in other types of cancers and discuss emerging areas of interest in the field.

Finally, two reviews debate about the impact of cancer cell activities on innate and cancer immunity. Cavillo-Rodriguez et al., explore the complex interactions between immunogenic cell death (ICD) induced by cancer therapies and the innate immune system, and address the next challenges in cancer treatment. cGAS-STING pathway and autophagy have been shown to be interrelated in innate immunity. Along the same line, Lu et al. summarize the recent findings of the cGAS-STING pathway and autophagy in cancer immunity and explore their interactions in this context, theorizing that strategies targeting these processes can be exploited for novel combination cancer immunotherapies.

Lastly, we would like to express our gratitude to all the authors who have contributed to this Research Topic, as well as to the reviewers for their remarkable commitment. We hope that the insights presented in this Research Topic will serve as a source of inspiration for those already working in the field of cancer immunotherapy, and as a fruitful and engaging read for those less familiar with this area of research.

## Author contributions

AC: Writing – original draft. MP-M: Writing – review & editing. VL: Writing – review & editing. DW: Writing – review & editing. CP: Writing – review & editing.

